# First documented extended-spectrum β-lactamase producing *Escherichia coli* renal abscess in Lebanon: a case report

**DOI:** 10.1097/MS9.0000000000004185

**Published:** 2025-10-27

**Authors:** Rabih Awad, Ahmad A El Lakis, Jana Kotaich, Ahmad El Issawi, Robert Harran

**Affiliations:** a Urology Department, Faculty of Medicine, Lebanese University, Beirut, Lebanon; b Faculty of Medicine, Lebanese University, Beirut, Lebanon; c MEDICA, Research Investigation, Hadath, Lebanon; d Urology Department, Sacré Coeur Hospital, Beirut, Lebanon

**Keywords:** abscess, child, ESBL, Escherichia coli, renal

## Abstract

**Introduction and importance:**

Renal abscesses in children are rare, and those caused by extended-spectrum β-lactamase (ESBL)-producing *Escherichia coli* are even rarer. This report presents the first case of an ESBL-producing *E. coli* renal abscess via a possible ascending route in a previously healthy child in Lebanon.

**Case presentation:**

A 10-year-old girl presented with high-grade fever, abdominal pain, and vomiting. Urine culture revealed an ESBL-producing *E. coli* infection, and imaging revealed multiple abscesses in the right kidney. Despite antibiotic treatment, the fever persisted, with abscess >3 cm requiring percutaneous drainage. After this procedure, the patient improved and was discharged with antibiotics. Follow-up imaging confirmed full resolution of all abscesses, and the voiding cystourethrogram was negative.

**Clinical discussion:**

Clinical diagnosis of a renal abscess in a previously healthy child without structural abnormalities or recent antibiotic use requires a high index of suspicion. Based on the literature and our case, we propose a diagnostic and management algorithm to guide evaluation and treatment.

**Conclusion:**

This case highlights the challenges of diagnosing and treating pediatric renal abscesses caused by ESBL-producing bacteria, marking the first reported case in Lebanon. The algorithm proposed is clinically useful but requires further validation in larger cohorts.

## Background

The World Health Organization (WHO) has identified antimicrobial-resistant bacteria (AMR) as one of the top 10 threats to global public health[1]. Over the years, this threat has extended to include more types of bacteria, particularly *Escherichia coli*, which have developed resistance to both oxyimino-cephalosporins and fluoroquinolones since the 2000s[1]. A notable antimicrobial-resistant pathogen is the extended-spectrum β-lactamase (ESBL)-producing *E. coli* ^[1,2]^. ESBL is a type of enzyme produced by specific bacteria that enables them to hydrolyze extended-spectrum cephalosporins^[2,3]^. As a result, these bacteria are resistant to beta-lactam antibiotics such as ceftazidime, cefotaxime, ceftriaxone, and oxyimino-monobactam, making their treatment extremely difficult^[2,3]^.

**HIGHLIGHTS**

- First pediatric renal abscess caused by ESBL-producing *Escherichia coli* reported in Lebanon.
- Occurred in a previously healthy child with no known risk factors via ascending route.
- Percutaneous drainage was required despite targeted antibiotics.
- Highlights rising antimicrobial resistance in the community.
- Proposed a diagnostic and management algorithm for pediatric abscess.

Renal abscesses in children are rare but one of the most severe forms of infectious kidney disease[4]. Delayed diagnosis commonly leads to poor therapeutic outcomes and severe renal complications^[4,5]^. Renal abscesses caused by ESBL-producing bacteria are extremely rare, with only two other documented pediatric cases^[5,6]^.

In Lebanon, surveillance studies have documented substantial prevalence of ESBL production among community and hospital isolates. For example, nationwide data indicate that about 32.3% of *E. coli* isolates produce ESBLs, and 29.2% of Klebsiella spp. isolates are ESBL producers[7]. In a community study of healthy infants, 49.6 % were colonized with ESBL-producing Enterobacteriaceae[8]. More recently, among Enterobacterales isolates resistant to extended-spectrum cephalosporins (2019–2020), ~30% were confirmed ESBL-producers, with *E. coli* accounting for nearly 78% of cases[9].

In this report, we present the first documented Lebanese pediatric case of a renal abscess due to community-acquired ESBL-producing *E. coli*. Beyond describing the clinical course, our objective is to propose a practical diagnostic and management algorithm to guide clinicians when facing similarly challenging infections. This case report has been reported in line with the SCARE checklist[10].

## Case presentation

A 10-year-old previously healthy girl presented to the emergency department after a one-day history of persistent high-grade fever accompanied by chills, diffuse abdominal pain, nausea, and vomiting. She had no history of urinary tract infections, urinary symptoms, fever of unknown origin, or prior surgeries. She reports no prior hospital admissions, and no recent antibiotics use. Upon examination, she appeared ill and exhibited decreased activity but with no signs of dehydration. Physical exam was unremarkable except for mild tenderness on the right flank. Vitals were remarkable for marked fever, with other parameters within normal limits.

**Figure 1.**
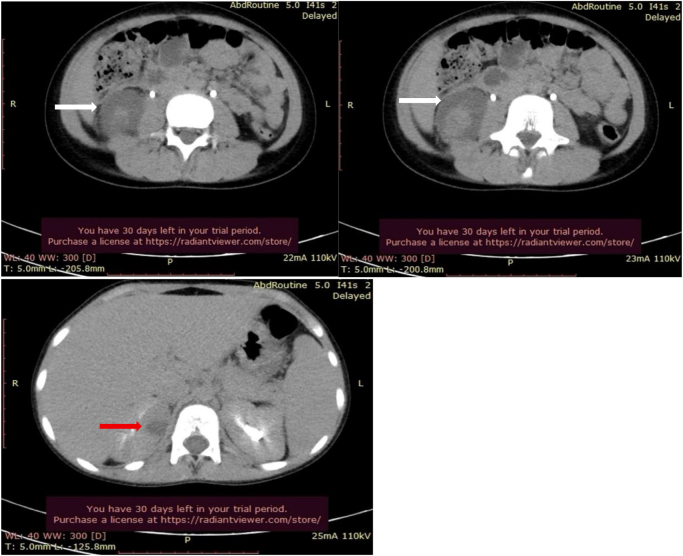
Abdominopelvic CT scan with IV contrast done revealed a 4-mm cortical micro abscess in the upper pole (red arrow) and a 4-cm abscess in the lower pole (white arrow).

The laboratory results revealed a hemoglobin level of 13.8 g/dL, creatinine level of 0.6 mg/dL, leukocyte count of 17 000/mm^3^, 81% neutrophils, and elevated C-reactive protein 190 mg/L. Urinalysis revealed 10–12 WBCs/hpf and positive leukocyte esterase. Additional workup showed normal chest X-ray and negative stool analysis with culture results. Ceftriaxone 2 g IV once daily (75 mg/Kg daily) was started but fever and abdominal pain persisted despite more than 48 hours of antibiotic treatment. Consequently after 2 days urine culture returned positive for ESBL *E. coli* (Table 1), along with laboratory results of leukocytes 15 000/mm^3^, neutrophils at 74%, and CRP at 68 mg/L As a result, ceftriaxone was discontinued, and Meropenem 500 mg IV three times daily (20 mg/kg/dose every 8 hours) was initiated. Patient remained febrile after 2 days on Meropenem, repeat urine analysis with culture was negative with other workups such as Widal and Wright also returning negative. Thus, we performed an abdominal and pelvic ultrasound which showed a hypoechoic lower pole lesion with no signs of obstruction or hydronephrosis, followed by an abdominopelvic CT scan with IV contrast revealed a 4 mm cortical micro abscess in the upper pole and a 4 cm abscess in the lower pole, with no signs of obstruction and no other findings (Fig. 1). Therefore, Meropenem was continued but at a maximum dose of 1 g intravenously three times per day.

**Table 1:** ESBL *E. coli* susceptibility in urine (S = susceptible, I = intermediate, R = resistant)

Antibiotics	*E. coli* > 100 000 CFU/ml
Amikacin	[S]
Amoxicillin	[R]
Amoxicillin/Clavulanic acid	[S]
Aztreonam	[S]
Cefaclor	[R]
Cefixime	[S]
Cefotaxime	[I]
Cefoxitin	[S]
Ceftazidime	[S]
Ceftriaxone	[I]
Cefuroxime	[R]
Doxycylin	[R]
Gentamycin	[S]
Imipenem	[S]
Meropenem	[S]
Nitrofurantoin	[S]
Piperacillin	[R]
Tobramycin	[S]
Trimethoprim/Sulfadiazine	[S]

**Figure 2.**
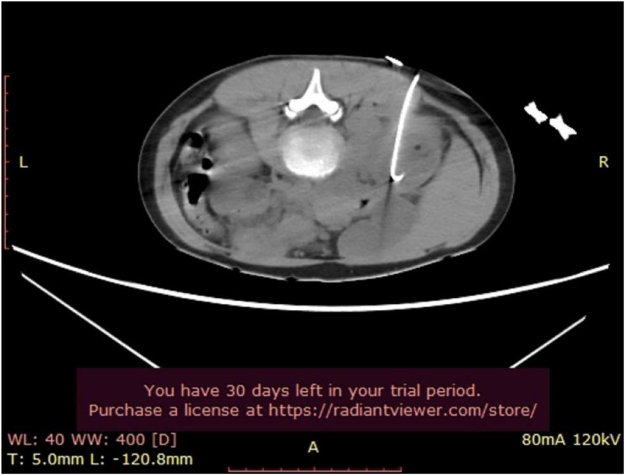
CT-guided drainage of the abscess: nephrostomy tube seen.

After 4 days on high-dose Meropenem, the persistence of fever and unchanging abscess on ultrasound with size >3 cm necessitated resorting to interventional management. Low-dose CT scan guided drainage of the abscess was performed by inserting an 8 Fr nephrostomy catheter, (Fig. 2) and 10 cc of pus was aspirated at the time of insertion with abscess culture results presented *E. coli* ESBL as seen on antibiogram (Table 2). The catheter was removed after 3 days of no more drainage, and the girl had no fever or complaints. The patient remained afebrile until she was discharged after a total of 1 week on maximum dose Meropenem and continued at home with a 2-week course of Ertapenem followed by 1 week of Ciprofloxacin, for which she was sensitive. One-month after the patient was completely cured, laboratory tests, urine analysis with culture, and voiding cystourethrogram and ultrasound all showed negative results. Structured day-by-day summary of symptoms, labs, imaging, interventions, and antibiotics are presented in Table 3.

**Table 2:** ESBL *E. coli* susceptibility in abscess (S = susceptible, I = intermediate, R = resistant)

Antibiotics	*E. coli* > 100 000 CFU/ml
Amikacin	[S]
Amoxicillin	[R]
Amoxicillin/Clavulanic acid	[S]
Aztreonam	[S]
Cefaclor	[R]
Cefixime	[S]
Cefotaxime	[I]
Cefoxitin	[S]
Ceftazidime	[S]
Ceftriaxone	[R]
Cefuroxime	[R]
Doxycylin	[R]
Gentamycin	[S]
Imipenem	[S]
Meropenem	[S]
Nitrofurantoin	[S]
Piperacillin	[R]
Tobramycin	[S]
Trimethoprim/Sulfadiazine	[S]

**Table 3:** Structured day-by-day summary of symptoms, labs, imaging, interventions, and antibiotics

Hospital day	Day 1	Day 3	Day 5	Day 9	Day 12	Day 13
Clinical events	High grade fever with diffuse abdominal pain & vomiting	Persisting fever & abdominal pain	Febrile with vague abdominal pain	Clinical improvement but still febrile	Afebrile stable vitals	Hemo-dynamically stable
						Discharged home
Labs	Hb: 13.8	WBC: 15 K	WBC: 10 K	WBC: 8 K	All labs within normal ranges Abscess cx ESBL *E. coli*	
	WBC: 17 K	CRP: 68	CRP: 290	CRP: 40		
	CRP: 190	Urine Cx: ESBL *E. coli*	Widal & Wright −ve			
	U/A + ve					
			Repeat U/A & urine Cx −ve			
			Blood cx –ve			
Antibiotics	Rocephine 2 g IV OD	Changed to Meropenem 500 mg IV TID	Meropenem increased to 1 g IV TID			
Imaging	Normal chest xray		Abdopelvic US: hypoechoic lower pole lesion, no obstruction	Abdopelvic US: imaging evidence of unchanging abscess >3 cm	No more drainage, nephrostomy removed	
			Abdopelvic CT: 4 mm cortical micro abscess in the upper pole and a 4 cm abscess in the lower pole	Low dose CT scan guided drainage of the abscess with nephrostomy		

CRP: C-reactive protein, cx: culture, OD: once daily, TID: 3× daily, U/A: urine analysis, −ve/ + ve: negative/positive result, US: ultrasound, WBC: white blood cells

## Discussion

The prevalence of community-acquired *E. coli* producing ESBL in children, has increased due to the rise of antimicrobial resistance[11]. To date, only two documented cases of renal abscesses caused by ESBL-producing *E. coli* in children exist (Table 4). One case involved a child who initially had a pan-sensitive *E. coli* abscess that was treated with ceftriaxone followed by 3 weeks of cefdinir and then, after recurrence, given trimethoprim-sulfamethoxazole. The symptoms did not improve, so CT scan revealed recurrence of the abscess but this time the organism was an ESBL-producing *E. coli* abscess. Despite treating with Meropenem 1000 mg three times no improvement was seen after 4 days, so percutaneous drainage done[6]. The second case involved a renal abscess due to hematogenous spread of ESBL-producing *E. coli*, and no drainage was needed, only antibiotic treatment was sufficient[5]. To our knowledge, this is the first reported case of a renal abscess caused by ESBL-producing *E. coli* via a possible ascending route in a previously healthy child in Lebanon.

**Table 4:** Published Pediatric ESBL *E. coli* renal/perirenal abscess cases: presentation, route, management, and outcomes

Case (year, country)	Age/Sex	Route (inferred)	Bacteremia	Management	Outcome	Reference
Community-acquired ESBL *E. coli* renal abscess complicating UTI (Urology, 2015, USA)	15 y/Female	Ascending (complicating UTI)	Not reported	Meropenem in-patient 1 week, 4 weeks Ertapenem on discharge	Clinical recovery reported	[6]
ESBL *E. coli* renal abscess with bacteremia (BMC Pediatrics, 2020, Japan)	5 y/Female	Likely hematogenous (blood culture first; urine initially negative; no VUR)	Yes	Meropenem; 3 weeks; no drainage	Abscess resolved; renal scarring on DMSA at 4 months	[5]
This report (Lebanon) – First pediatric ESBL renal abscess in Lebanon	10 y/Female	Likely ascending (positive urine culture; negative blood culture; normal VCUG)	No (blood culture negative)	Meropenem in-patient 10 days; 2 weeks Ertapenem + 1 week Ciprofloxacin on discharge	Full clinical and radiologic resolution at 1 month	Current case report

Renal abscesses may develop through two primary mechanisms, pathogens can either enter through a complicated urinary tract infection, ascending route, or spread via the bloodstream, hematogenous route^[12,13]^. In pediatric population, this condition is more frequently associated with urological abnormalities such as vesicoureteral reflux, calyceal diverticulum, ureteropelvic junction obstructions, and urolithiasis[13]. Additionally, it is crucial to note that a structural urinary tract abnormality is not always a required condition for the formation of a renal abscess in children^[12,13]^.

In our case, the patient had a positive urine and abscess culture with a negative blood culture and a normal voiding cystourethrogram (VCUG). This suggests a probable ascending route without a prerequisite structural abnormality or vesicourethral reflux despite negative history of recent hospitalization or antibiotic use. We could not determine the exact source of the infection, but this case highlights the potential rise in antimicrobial resistance outside hospital settings, even among healthy pediatric patients with no apparent risk factors. Possible sources of infection include community colonization from contaminated water sources, particularly in countries with sewage issues; household exposure from colonized individuals with prior antibiotic usage; and unregulated use of antibiotics in agriculture to control diseases in crops and livestock, which aids in the spread of ESBL genes^[1,14]^. It is worth mentioning that all the proposed sources are only suggestions when explanations are unclear, but caution should be taken in such framing.

Clinical diagnosis of renal abscesses is extremely difficult, especially in children, due to the nonspecific symptoms exhibited by these patients^[13,15]^. A triad of fever, nausea and vomiting, and flank pain are the most common symptoms, in addition to nonspecific laboratory results such as elevated CRP, abnormal urine analysis, and leukocytosis^[13,15]^. One of the most common cardinal symptoms is febrile episodes that persist despite appropriate antibiotic treatment which should raise suspicion for a complicated urinary tract infection (UTI) including a renal abscess[15]. In our case, the patient presented with the mentioned triad with abnormal laboratory results. She was initially started on a broad-spectrum cephalosporin antibiotic treatment for a suspected UTI. However, similar to the other previous two cases, after 2 days of persistent fever, the patient was switched to Meropenem following cultures that yielded ESBL-producing *E. coli*. The patient started on low dose of Meropenem and then switched to the maximum dose when she had fever even on antibiotic.

Further radiological evaluation is warranted when a complicated UTI is suspected[16]. Ultrasound is the initial screening method; however, it is widely accepted that a CT scan is necessary to confirm the diagnosis of a renal abscess^[12,13,15,16]^. According to the literature, pediatric renal abscesses that are small (≤3 cm) can be managed conservatively, whereas larger abscesses (>3 cm) may require percutaneous drainage if fever persists despite the use of culture-specific antibiotics^[13,17]^. In our case, the initial ultrasound was inconclusive, necessitating a CT scan, which confirmed the presence of a 4 cm renal abscess. After 4 days of targeted high-dose antibiotic treatment with persistent fever, and no regression of the abscess, a percutaneous drainage was performed.

While our case might suggest a rise in community-acquired ESBL infections in Lebanon, broader conclusions require caution. The high ESBL colonization rates in Lebanese infants (nearly 50 %)[8] and the ~33 % prevalence in urinary isolates indicate that our case fits within a broader reservoir of resistance[18]. However, pediatric renal abscesses caused by ESBL organisms remain extremely rare. Our case should be considered a sentinel event rather than definitive evidence of a regional surge. Therefore, this study is limited by its single-center design predisposing it to selection bias which hinders generalizability. Moreover, other limitations are the potential sources of the ESBL strain which could not be fully explored; thus, findings should be interpreted with caution.

**Figure 3.**
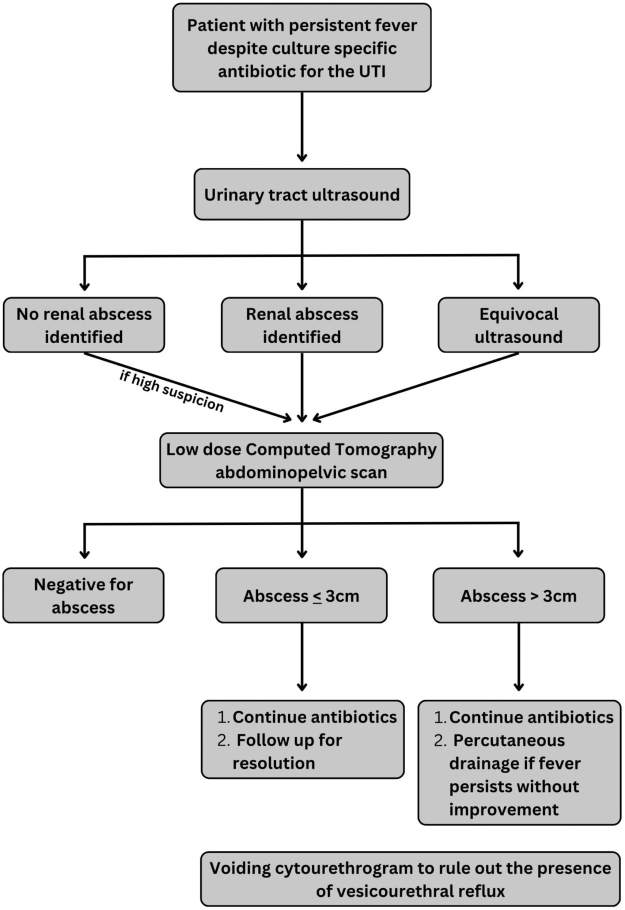
Diagnostic and management algorithm for pediatric renal abscess.

This case should alert physicians to maintain a high level of suspicion when encountering pediatric patients with persistent fever. Based on the literature review and the two previously documented cases, we propose the following diagnostic and management algorithm for pediatric renal abscess (Fig. 3). Nevertheless, this proposed pathway remains preliminary and requires validation in larger pediatric cohorts to avoid overgeneralization.

## Conclusion

This case highlights the challenges in diagnosing and treating a previously healthy child, with no history of antibiotic therapy, who developed an ESBL-producing *E. coli* renal abscess. This finding underscores the importance of responsible antibiotic stewardship to mitigate the growing risk of community-acquired ESBL-producing organisms even outside hospital settings in risk-free individuals.

The delayed response to cephalosporins and the eventual need for drainage in our patient directly demonstrate how ESBL resistance complicates clinical management. While our findings align with the high prevalence of ESBL colonization and resistance documented in Lebanon^[8,9,18,19]^, pediatric ESBL renal abscesses remain exceedingly rare. Thus, this report should be viewed as a sentinel case rather than evidence of a regional surge.

The diagnostic and management algorithm we propose is clinically useful but requires further validation in larger cohorts. Treatment decisions should be guided by the patient’s clinical improvement, with options ranging from antibiotic therapy alone to a combination of antibiotics and percutaneous drainage. In our case, antibiotic treatment alone was insufficient, so percutaneous drainage was performed.

## Data Availability

All data generated or analyzed during this study are included in this published article.
